# Supplementary material for: American black bear (Ursus americanus) as a potential host for Campylobacter jejuni
Source: PLoS One. 2025 Sep 9;20(9):e0331559. doi: 10.1371/journal.pone.0331559 (PMC12419602; doi:10.1371/journal.pone.0331559)
Supplement: S1 Table — (PDF) [file pone.0331559.s001.pdf]

Supplementary Table 1: Strains used for comparison

GLOBAL

Strain	Taxon	source	location	Year	ST	CC	GenBank accession #
SKBC1	*Cjj*	black bear feces	US: North Carolina	2014	222	206	CP125383
SKBC102	*Cjj*	black bear feces	US: North Carolina	2016	45	45	CP125387
SKBC25	*Cjj*	black bear feces	US: North Carolina	2015	10624	179	CP125388
SKBC3	*Cjj*	black bear feces	US: North Carolina	2014	7630	n/a	CP125394
SKBC41	*Cjj*	black bear feces	US: Virginia	2015	45	45	CP125386
SKBC5	*Cjj*	black bear feces	US: North Carolina	2014	10620	n/a	CP125391
SKBC57	*Cjj*	black bear feces	US: North Carolina	2016	21	21	CP125396
SKBC9	*Cjj*	black bear feces	US: Georgia	2014	10501	n/a	CP125390
SKBC94	*Cjj*	black bear feces	US: North Carolina	2016	682	682	CP125395
LMG 9217	*Cjj*	human stool	Belgium	1986	443	443	AIOO010000000
1997-1	*Cjj*	human stool	USA	1997	658	658	AIOT010000000
140-16	*Cjj*	cattle, stool	USA	Unk.	5161	61	AIPF01000000
NCTC 11168	*Cjj*	human stool	UK: England	Unk.	43	21	AL111168
1336	*Cjj*	water	Unknown	Unk.	841	n/a	CM000854
414	*Cjj*	bank vole	Unknown	Unk.	3704	n/a	CM000855
RM1221	*Cjj*	chicken meat	USA: California	1997	354	354	CP000025
81-176	*Cjj*	human stool	US: Minnesota	1982	604	42	CP000538
81116	*Cjj*	human stool	UK: England	1981	267	283	CP000814
IA3902	*Cjj*	sheep, aborted placenta	US: Iowa	2009	8	21	CP001876
M1	*Cjj*	human stool	UK: England	2010	137	45	CP001900
S3	*Cjj*	Unknown	Unknown	Unk.	354	354	CP001960
PT14	*Cjj*	human stool	UK: England	Unk.	50	21	CP003871
R14	*Cjj*	chicken	UK: England	Unk.	356	353	CP005081
CG8421	*Cjj*	human stool	Thailand: Bangkok	Unk.	1919	52	CP005388
00-2426	*Cjj*	human stool	Canada	2000	21	21	CP006708
F38011	*Cjj*	human stool	Unknown	Unk.	3644	n/a	CP006851
M129	*Cjj*	human stool	Unknown	1990	353	353	CP007749
D42a	*Cjj*	chicken cecum	USA	2007	21	21	CP007751
MTVDSCj20	*Cjj*	chicken cecum	US: Michigan	2013	8785	45	CP008787
YH001	*Cjj*	beef liver	US: Pennsylvania	2014	806	21	CP010058

01-1512	*Cjj*	human stool	Canada	2001	8	21	CP010072
00-0949	*Cjj*	human stool	Canada	2000	8	21	CP010301
00-1597	*Cjj*	human stool	Canada	2000	930	n/a	CP010306
00-6200	*Cjj*	human stool	Canada	2000	806	21	CP010307
RM3196	*Cjj*	human stool	South Africa: Capetown	1996	362	362	CP012690
RM1285	*Cjj*	chicken meat	USA: California	1997	22	22	CP015209
14980A	*Cjj*	turkey feces	US: North Carolina	2014	1839	n/a	CP017029
MTVDSCj07	*Cjj*	chicken cecum	US: Michigan	2013	8789	21	CP017031
MTVDSCj13	*Cjj*	chicken cecum	US: Michigan	2013	460	460	CP017032
MTVDSCj16	*Cjj*	chicken cecum	US: Michigan	2013	1911	n/a	CP017033
RM3420	*Cjj*	human stool	Canada	1981	41	41	CP017456
ATCC 35925	*Cjj*	pigeon	Sweden	1984	5843	n/a	CP020045
LDG17f	*Cjj*	human stool	Czechia	2016	464	464	CP040015
9090	*Cjj*	human stool	Slovenia	2009	50	21	CP040016
NADC 20827	*Cjj*	turkey feces	US: Iowa	2005	1212	607	CP045048
NCTC 11951^{T}	*Cjd*	human blood	Unknown	1986	62	n/a	LR134359
269.97	*Cjd*	human blood	South Africa: Capetown	1997	1845	n/a	CP000768
NCTC 11924	*Cjd*	human blood	Unknown	1999	-	n/a	LR134530
NCTC 11925	*Cjd*	human blood	Australia	1988	8767	n/a	LS483295
HF5-4A-4	*Cj*	farm environment	UK	2012	861	21	CP007188
HF5-5-1	*Cj*	farm environment	UK	2012	45	45	CP007189
HF5-7-1	*Cj*	farm environment	UK	2012	45	45	CP007190
NS4-1-1	*Cj*	farm environment	UK	2012	21	21	CP007191
NS4-5-1	*Cj*	farm environment	UK	2012	21	21	CP007192
NS4-9-1	*Cj*	farm environment	UK	2012	21	21	CP007193
CJ677CC539	*Cj*	human stool	Finland	1996	794	677	CP010457
FORC_046	*Cj*	human stool	South Korea: Seoul	2016	22	22	CP017229
BCW_6920	*Cj*	cow, abortion	Unknown	2011	8	21	CP017673
NCTC 12662	*Cj*	Unknown	UK	1992	5843	n/a	CP019965
YH002	*Cj*	calf liver	US: Pennsylvania	2014	982	21	CP020776
12567	*Cj*	chicken	UK: England	2005	53	21	CP028909
NCTC 12660	*Cj*	chicken	UK: England	2005	21	21	CP028910
NCTC 12661	*Cj*	human stool	Sweden	1985	5843	n/a	CP028911
NCTC 12664	*Cj*	chicken	UK: England	1992	50	21	CP028912
FORC_083	*Cj*	chicken meat	South Korea: Seoul	2017	6849	354	CP028933

SCJK2	*Cj*	mouse	South Korea: Gangwon	2017	8388	n/a	CP038862
YH003	*Cj*	chicken meat	US: Pennsylvania	2014	353	353	CP041584
D33a	*Cj*	chicken cecum	US: Arizona	2003	459	42	CP058293
A14a	*Cj*	chicken cecum	US: Kansas	2003	1212	607	CP058295
A9a	*Cj*	chicken cecum	US: Kansas	2003	2827	460	CP058299
OXC6271	*Cj*	human stool	UK: England	2011	508	508	CUID01000000
OXC6291	*Cj*	human stool	UK: England	2011	2030	257	CUIW01000000
OXC6306	*Cj*	human stool	UK: England	2011	273	206	CUJO01000000
OXC6356	*Cj*	human stool	UK: England	2011	464	464	CULI01000001
OXC6392	*Cj*	human stool	UK: England	2011	574	574	CUMV01000001
OXC6433	*Cj*	human stool	UK: England	2011	573	573	CUOL01000001
OXC6484	*Cj*	human stool	UK: England	2011	403	403	CUQC01000001
GP012	*Cj*	guinea pig stool	Peru: Iquitos	2019	10316	n/a	JACRSF000000000
BCW_4460	*Cj*	rhesus macaque feces	USA: California	2015	9259	177	MKAJ01000001
BCW_5913	*Cj*	rhesus macaque feces	USA: California	2016	6629	179	MKES01000001

GEOGRAPHICALLY LOCAL *Campylobacter jejuni*
(Eastern USA; especially Georgia (GA), North Carolina (NC), Virginia(VA)):

Isolate	Source	State	Year	ST	CC	PubMLST id	NCBI Biosample/SRA_accession #
PNUSAC007067	human stool	n/a	n/a	2524	179	89537	n/a
PNUSAC007529	human stool	n/a	n/a	10501	n/a	89602	n/a
FSIS11811613	chicken offal or meat	VA	2018	222	206	91623	n/a
FSIS1702328	chicken offal or meat	GA	2017	45	45	92249	SAMN07410381; SRS2380429
FSIS1609690	chicken offal or meat	NC	2016	50	21	92472	SAMN06256283; SRS1938321
FSIS1607586	chicken offal or meat	GA	2016	2083	n/a	92497	SAMN05763277; SRS1689518
FSIS1609037	chicken offal or meat	GA	2016	353	353	92504	SAMN06140436; SRS1858539
FSIS1607764	chicken offal or meat	GA	2016	6091	n/a	92518	SAMN05833071; SRS1717570
FSIS1607591	chicken offal or meat	GA	2016	2083	n/a	92530	SAMN05799409; SRS1707549
FSIS1607633	chicken offal or meat	GA	2016	51	443	92535	SAMN05799436; SRS1707552
FSIS11704849	chicken offal or meat	GA	2017	222	206	92624	SAMN08224441; SRS2780950
FSIS1710104	chicken	GA	2017	222	206	92662	SAMN06328364; SRS1974250
FSIS1609201	chicken	GA	2016	51	443	92695	SAMN06179997; SRS1879691
FSIS1608564	chicken	GA	2016	460	460	92734	SAMN06046130; SRS1813091

FSIS1608026	chicken offal or meat	GA	2016	353	353	92744	SAMN05945123; SRS1761697
FSIS1609374	chicken offal or meat	GA	2016	50	21	92750	SAMN06210480; SRS1902216
FSIS1609034	chicken offal or meat	NC	2016	3510	353	92764	SAMN06140433; SRS1858531
FSIS1608861	chicken offal or meat	VA	2016	353	353	92770	SAMN06127095; SRS1847025
FSIS1608913	chicken offal or meat	GA	2016	50	21	92795	SAMN06127104; SRS1847027
FSIS1608569	chicken offal or meat	VA	2016	353	353	92810	SAMN06046135; SRS1813101
FSIS1608023	chicken	VA	2016	3510	353	92820	SAMN05945120; SRS1761716
FSIS1501392	chicken	NC	2015	2083		92873	SAMN03785200; SRS969568
FSIS1501558	chicken offal or meat	GA	2015	443	443	92876	SAMN03850816; SRS985870
FSIS1501391	chicken offal or meat	GA	2015	939	353	92878	SAMN03785199; SRS969566
FSIS1609045	chicken offal or meat	GA	2016	404	353	93114	SAMN06140444; SRS1858547
FSIS1608758	chicken	GA	2016	50	21	93123	SAMN06127133; SRS1847102
FSIS1608911	chicken offal or meat	GA	2016	454	21	93129	SAMN06127102; SRS1847040
FSIS1608858	chicken offal or meat	GA	2016	464	464	93132	SAMN06127092; SRS1847029
FSIS1608345	chicken offal or meat	GA	2016	939	353	93154	SAMN06015823; SRS1797969
FSIS1608019	chicken	NC	2016	353	353	93170	SAMN05945116; SRS1761704
FSIS1608020	chicken offal or meat	VA	2016	3515	353	93175	SAMN05945117; SRS1761722
FSIS1607914	chicken offal or meat	GA	2016	354	354	93176	SAMN05928212; SRS1753175
FSIS1607616	chicken	NC	2016	3736	353	93183	SAMN05799424; SRS1707551
FSIS1607623	chicken offal or meat	GA	2016	4370	353	93186	SAMN05799430; SRS1707521
FSIS1501400	chicken offal or meat	GA	2015	939	353	93200	SAMN03785205; SRS969573
FSIS1504340	chicken	GA	2015	51	443	93345	SAMN04267339; SRS1162925
FSIS1500509	chicken	GA	2013	939	353	93346	SAMN03897488; SRS1010270
FSIS1504347	chicken offal or meat	VA	2015	464	464	93348	SAMN04267345; SRS1162923
FSIS1501385	chicken offal or meat	GA	2015	353	353	93350	SAMN03785194; SRS969558
FSIS1609206	chicken offal or meat	GA	2016	454	21	93657	SAMN06180002; SRS1879683
FSIS1609368	chicken offal or meat	GA	2016	51	443	93659	SAMN06256270; SRS1938334
FSIS1709950	chicken offal or meat	NC	2016	3736	353	93686	SAMN06276939; SRS1945030
FSIS1609187	chicken offal or meat	GA	2016	10703	353	93690	SAMN06179983; SRS1879695
FSIS1609191	chicken offal or meat	VA	2016	353	353	93695	SAMN06179987; SRS1879692
FSIS1609538	chicken offal or meat	NC	2016	4370	353	93944	SAMN06602732; SRS2049484
FSIS1609040	chicken offal or meat	VA	2016	3510	353	93958	SAMN06140439; SRS1858554
FSIS1609190	chicken offal or meat	GA	2016	10751	607	93959	SAMN06179986; SRS1879698
FSIS1608029	chicken offal or meat	GA	2016	50	21	93961	SAMN05945126; SRS1761711
FSIS1608091	chicken offal or meat	GA	2016	3510	353	93962	SAMN05945140; SRS1761712

FSIS1607926	chicken	VA	2016	4370	353	93968	SAMN05928223; SRS1753158
FSIS1607848	chicken offal or meat	GA	2016	353	353	93969	SAMN05900945; SRS1742329
FSIS1607760	chicken offal or meat	GA	2016	4376	353	93977	SAMN05833067; SRS1717579
FSIS1607701	chicken offal or meat	VA	2016	353	353	93984	SAMN05833099; SRS1717486
FSIS1607705	chicken offal or meat	GA	2016	939	353	93985	SAMN05833103; SRS1717495
FSIS1607620	chicken offal or meat	GA	2016	2132	353	93988	SAMN05799427; SRS1707438
FSIS1607619	chicken offal or meat	NC	2016	10520	353	93990	SAMN05799426; SRS1707447
FSIS1504326	chicken offal or meat	GA	2015	6091	n/a	94009	SAMN04267325; SRS1162916
FSIS1504342	chicken offal or meat	NC	2015	3694	48	94012	SAMN04267341; SRS1162920
FSIS1504348	chicken offal or meat	NC	2015	1212	607	94017	SAMN04267346; SRS1163359
FSIS1504331	chicken offal or meat	GA	2015	10221	353	94021	SAMN04267330; SRS1163509
FSIS1501091	chicken offal or meat	NC	2015	2829	353	94028	SAMN03795224; SRS973092
FSIS1501559	chicken offal or meat	GA	2015	6091	n/a	94033	SAMN03850817; SRS985871
FSIS1501560	chicken offal or meat	GA	2015	50	21	94037	SAMN03850818; SRS985873
FSIS11816823	chicken	GA	2018	137	45	94752	SAMN10881670; SRS4337209
FSIS21923364	chicken offal or meat	NY	2019	2524	179	94767	SAMN10880123; SRS4333512
CVM N55904	chicken offal or meat	MO	2017	10501	n/a	97941	SAMN06660919; SRS2099146
FSIS1606899	cattle	TN	2016	2524	179	98512	SAMN05366799, SRS1548480
FSIS1607233	cattle	NC	2016	918	48	99391	SAMN05558641; SRS1609336
FSIS1607149	cattle	NC	2016	3736	353	99419	SAMN05505603; SRS1598342
FSIS1606848	cattle	NC	2016	982	21	99559	SAMN05301270; SRS1529695
FSIS1606461	cattle	GA	2016	61	61	99598	SAMN05190020; SRS1475011
FSIS1606460	cattle	GA	2016	8	21	99604	SAMN05190019; SRS1475009
FSIS1606459	cattle	VA	2016	42	42	99607	SAMN05190018; SRS1475008
FSIS1606467	cattle	VA	2016	6673	257	99673	SAMN05190026; SRS1475017
FSIS1606252	cattle	GA	2016	61	61	99810	SAMN04625697; SRS1382924
FSIS1605855	cattle	GA	2016	8	21	99833	SAMN04524182; SRS1316544
FSIS1606151	cattle	NC	2016	607	607	99840	SAMN04600084; SRS1371155
FSIS1606106	cattle	NC	2016	61	61	99842	SAMN04600066; SRS1371151
FSIS1605890	cattle	NC	2016	21	21	99904	SAMN04526075; SRS1318878
FSIS1605939	cattle	NC	2016	982	21	99928	SAMN04537006; SRS1327433
FSIS1605623	cattle	NC	2016	459	42	100060	SAMN04461472; SRS1278043
FSIS1605801	cattle	GA	2016	61	61	100061	SAMN04508250; SRS1307320
FSIS1605627	cattle	NC	2016	1244	61	100074	SAMN04461490; SRS1277875
FSIS1607321	cattle	GA	2016	21	21	100701	SAMN05578358; SRS1620915

FSIS11807237	chicken	VA	2017	45	45	101041	SAMN08437409; SRS2898277
FSIS1607526	cattle	NC	2016	929	257	101272	SAMN05763253; SRS1689521
FSIS1609265	cattle	VA	2016	806	21	101332	SAMN06179687; SRS1878018
FSIS1504735	cattle	GA	2015	1244	61	101494	SAMN04452114; SRS1272300
FSIS1502230	cattle	NC	2015	61	61	101498	SAMN04388419; SRS1240223
FSIS1503662	cattle	GA	2015	8	21	101507	SAMN04388448; SRS1240204
FSIS1501831	cattle	NC	2015	982	21	101538	SAMN04320879; SRS1192310
FSIS1501958	cattle	NC	2015	61	61	101539	SAMN04320886; SRS1192366
FSIS1504630	cattle	NC	2015	42	42	101554	SAMN04195060; SRS1121793
FSIS1501333	cattle	NC	2015	8	21	101567	SAMN04091194; SRS1071596
FSIS1609158	chicken	GA	2016	353	353	101870	SAMN06165733; SRS1871244
FSIS1605533	cattle	NC	2015	61	61	102020	SAMN04444345; SRS1266750
FSIS1503058	cattle	GA	2015	806	21	102021	SAMN04388427; SRS1240219
FSIS1504631	cattle	NC	2015	21	21	102067	SAMN04195061; SRS1121792
FSIS1501427	cattle	GA	2015	982	21	102223	SAMN04027116; SRS1054389
FSIS1710033	cattle	GA	2016	806	21	103340	SAMN06276449; SRS1945037
FSIS1710042	cattle	NC	2016	1244	61	103348	SAMN06276453; SRS1945001
FSIS1701165	cattle	GA	2017	22	22	103991	SAMN06915353; SRS2172035
FSIS1710460	cattle	GA	2016	61	61	104131	SAMN06346617; SRS1991268
FSIS1608309	cattle	TN	2016	10501		104166	SAMN06046063; SRS1812533
FSIS1607683	cattle	VA	2016	806	21	104208	SAMN05833081; SRS1717485
FSIS1607693	chicken	GA	2016	51	443	104218	SAMN05833091; SRS1717476
FSIS1501977	swine	VA	2015	21	21	104332	SAMN04320901; SRS1192377
FSIS1501832	cattle	NC	2015	806	21	104333	SAMN04320880; SRS1192304
FSIS1504858	cattle	NC	2015	982	21	104343	SAMN04229854; SRS1144016
FSIS1501323	swine	NC	2015	13296	354	104370	SAMN04091185; SRS1071587
SFBRC-1	environmental water	GA	2012	3889	179	106318	n/a
SFBRC-2	environmental water	GA	2012	61	61	106319	n/a
SFBRC-28	environmental water	GA	2013	2524	179	106336	n/a
SFBRC-29	environmental water	GA	2013	2524	179	106337	n/a
SFBRC-36	environmental water	GA	2013	7943		106344	n/a
SFBRC-41	environmental water	GA	2013	699	692	106347	n/a
SFBRC-42	environmental water	GA	2013	2866		106348	n/a
SFBRC-52	environmental water	GA	2013	3889	179	106350	n/a

SFBRC-53	environmental water	GA	2013	692	692	106351	n/a
SFBRC-57	environmental water	GA	2013	692	692	106352	n/a
SFBRC-59	environmental water	GA	2013	7943	n/a	106353	n/a
SFBRC-60	environmental water	GA	2013	7949	n/a	106354	n/a
SFBRC-66	environmental water	GA	2013	7949	n/a	106355	n/a
FSIS11922763	chicken	KY	2019	10501	n/a	120341	SAMN12349472; SRS5145307
FSIS11923010	swine	VA	2019	464	464	120435	SAMN12345917; SRS5144437
FSIS12027905	swine	NC	2019	45	45	122671	SAMN14737992; SRS6540860
FSIS12106873	cattle	PA	2021	682	682	126827	SAMN23493042; SRS11178343
FSIS12142080	turkey	VA	2021	45	45	128966	SAMN21463028; SRS10189879
FSIS12142452	cattle	NC	2021	45	45	129106	SAMN21337215; SRS10053674
FSIS21926450	chicken offal or meat	GA	2019	45	45	134474	SAMN13708426; SRS5931241
FSIS22207643	chicken offal or meat	VA	2022	48	48	138360	SAMN27522664; SRS12574200
FSIS11808604	cattle	NC	2018	21	21	102857	SAMN08816081; SRS3106598
FSIS12034998	cattle	NC	2020	21	21	125437	SAMN16811271; SRS7723740
FSIS12208588	chicken carcass	NC	2021	222	206	129596	SAMN25159901; SRS11733377
FSIS12322565	raw intact chicken	AL	2023	n/a	n/a	n/a	SAMN35685631; SRS17944688
FSIS1502371	cattle	NJ	2015	45	45	101541	SAMN04120349; SRS1181114
PNUSAC000204	human stool	n/a	n/a	6647	49	85526	SAMN04339734; SRS1211470
PNUSAC004706	human stool	n/a	n/a	10446	45	82515	SAMN09292332; SRS3363973
PNUSAC005155	human stool	n/a	n/a	45	45	81938	SAMN09665077; SRS3549084
PNUSAC010171	human stool	n/a	n/a	n/a	n/a	n/a	SAMN12585128; SRS5273649
FSIS22026997	raw intact chicken	GA	2020	222	206	134728	SAMN14085345; SRS6133342
FSIS31902241	chicken carcass	GA	2019	45	45	140604	SAMN12163344; SRS5043817
NC_C3496	water	NC	2009	n/a	n/a	n/a	SAMN08168977; SRS3211689
