# Supplementary material for: American black bear (Ursus americanus) as a potential host for Campylobacter jejuni
Source: PLoS One. 2025 Sep 9;20(9):e0331559. doi: 10.1371/journal.pone.0331559 (PMC12419602; doi:10.1371/journal.pone.0331559)
Supplement: S2 Table — (PDF) [file pone.0331559.s002.pdf]

Supplementary Table 2: *C. jejuni* Strains in PubMLST that share four alleles with either SKBC9 or SKBC25

Strain	Location[a]	Year	source	*aspA*	*glnA*	*gltA*	*glyA*	*pgm*	*tkt*	*uncA*	ST	CC[b]
06-1507	Canada:Quebec	2006	env. water	1	6	61	244	40	32	3	2524	179
007A-0395	Canada:Ontario	2005	env. water	1	6	61	176	40	32	3	4380	179
BICO 301 (14)	USA:Georgia	2005	env. water	1	6	61	244	40	32	3	2524	179
NORO 513 (83a)	USA:Georgia	2007	env. water	1	6	61	244	40	32	3	2524	179
NORO 115 (38a)	USA:Georgia	2008	env. water	1	6	61	244	40	32	3	2524	179
SFBRC-12	USA:Georgia	2012	env. water	1	6	61	244	40	32	3	2524	179
SFBRC-48	USA:Georgia	2013	env. water	1	6	61	244	40	32	3	2524	179
PNUSAC009587	USA	Unk.	human	1	6	61	244	40	32	3	2524	179
PNUSAC009549	USA	Unk.	human	1	6	61	244	40	32	3	2524	179
PNUSAC001967	USA:HHS_region 2	2016	human	1	6	61	244	40	32	3	2524	179
PNUSAC005720	USA:HHS_region 5	2018	human	1	6	61	176	40	32	3	4380	179
PNUSAC005568	USA:HHS_region 4	2018	human	1	6	61	244	40	32	3	2524	179
PNUSAC005579	USA:HHS_region 3	2018	human	1	6	61	244	40	32	3	2524	179
PNUSAC005116	USA:HHS_region 3	2018	human	1	6	61	244	40	32	3	2524	179
PNUSAC009231	USA	Unk.	human	1	6	61	244	40	32	3	2524	179
PNUSAC002170	USA:HHS_region 2	2016	human	1	6	61	244	40	32	3	2524	179
PNUSAC003437	USA:HHS_region 5	2017	human	12	6	61	147	261	32	3	10501	NA
PNUSAC004034	USA:Wyoming	2011	cattle	1	6	61	244	40	32	3	2524	179
PNUSAC001646	USA:HHS_region 1	2017	human	1	6	61	176	40	32	3	4380	179
PNUSAC001432	USA:HHS_region 3	2016	human	12	6	61	244	40	32	3	10537	179
PNUSAC004020	USA:HHS_region 5	2017	human	1	6	61	176	40	32	3	4380	179
PNUSAC003948	USA:HHS_region 4	2017	human	1	6	61	244	40	32	3	2524	179
PNUSAC003780	USA:HHS_region 3	2017	human	1	6	61	244	40	32	3	2524	179
PNUSAC003495	USA:HHS_region 3	2017	human	1	6	61	244	40	32	3	2524	179
PNUSAC002060	USA:HHS_region 8	2017	human	1	6	61	244	40	32	3	2524	179
PNUSAC003351	USA:HHS_region 3	2017	human	1	6	61	244	40	32	3	2524	179
PNUSAC002955	USA:HHS_region 5	2017	human	1	6	61	244	40	32	3	2524	179
PNUSAC007552	USA	Unk.	human	1	6	61	244	40	32	3	2524	179
PNUSAC007662	USA	Unk.	human	1	6	61	244	40	32	3	2524	179
PNUSAC007820	USA	Unk.	human	1	6	61	244	40	32	3	2524	179
PNUSAC008247	USA	Unk.	human	1	6	61	244	40	32	3	2524	179
PNUSAC007856	USA	Unk.	human	1	6	61	244	40	32	3	2524	179
PNUSAC007714	USA	Unk.	human	1	6	61	244	40	32	3	2524	179
PNUSAC007690	USA	Unk.	human	1	6	61	244	40	32	3	2524	179

PNUSAC007067	USA	Unk.	human	1	6	61	244	40	32	3	2524	179
PNUSAC007535	USA	Unk.	human	1	6	61	176	40	32	3	4380	179
PNUSAC007529	USA	Unk.	human	12	6	61	147	261	32	3	10501	NA
PNUSAC007290	USA	Unk.	human	12	6	61	147	261	32	3	10501	NA
PNUSAC007217	USA	Unk.	human	1	6	61	244	40	32	3	2524	179
PNUSAC007216	USA	Unk.	human	1	6	61	244	40	32	3	2524	179
PNUSAC007165	USA	Unk.	human	1	6	61	244	40	32	3	2524	179
PNUSAC007116	USA	Unk.	human	1	6	61	244	40	32	3	2524	179
PNUSAC006882	USA:HHS_region 5	2018	human	1	6	61	176	40	32	3	4380	179
PNUSAC006826	USA:HHS_region 1	2018	human	1	6	61	244	40	32	3	2524	179
PNUSAC006211	USA:HHS_region 3	2018	human	1	6	61	176	40	32	3	4380	179
PNUSAC006032	USA:HHS_region 5	2018	human	1	6	61	244	40	32	3	2524	179
PNUSAC006234	USA:HHS_region 4	2018	human	1	6	61	244	40	32	3	2524	179
PNUSAC006588	USA:HHS_region 4	2018	human	1	6	61	244	40	32	3	2524	179
PNUSAC006687	USA:HHS_region 5	2018	human	1	6	61	244	40	32	3	2524	179
PNUSAC006589	USA:HHS_region 5	2018	human	1	6	61	176	40	32	3	4380	179
FSIS21720490	USA:Michigan	2017	chicken	1	6	61	244	40	32	3	2524	179
FSIS21923364	USA:New York	2019	chicken	1	6	61	244	40	32	3	2524	179
CVM N17C624	USA:Texas	2017	chicken	1	6	61	244	40	32	3	2524	179
CVM N55904	USA:Missouri	2015	chicken	12	6	61	147	261	32	3	10501	NA
CVM N18C002	USA:California	2018	turkey	1	6	61	176	40	32	3	4380	179
FSIS1606899	USA:Tennessee	2016	cattle	1	6	61	244	40	32	3	2524	179
FSIS11812606	USA:Iowa	2018	chicken	1	6	61	176	40	32	3	4380	179
FSIS1608309	USA:Tennessee	2016	cattle	12	6	61	147	261	32	3	10501	NA
**SKBC9**	**USA:Georgia**	**2014**	**black bear**	12	6	61	147	261	32	3	10501	NA
**SKBC25**	**USA:North Carolina**	**2015**	**black bear**	1	6	61	244	797	32	3	10624	179
SFBRC-28	USA:Georgia	2013	env. water	1	6	61	244	40	32	3	2524	179
SFBRC-29	USA:Georgia	2013	env. water	1	6	61	244	40	32	3	2524	179
SFBRC-32	USA:Georgia	2013	env. water	1	6	61	244	40	32	3	2524	179
FSIS11922763	USA:Kentucky	2019	chicken	12	6	61	147	261	32	3	10501	NA
FSIS11926407	USA:Ohio	2019	turkey	1	6	61	244	40	32	3	2524	179
FSIS12028294	USA:Arkansas	2020	chicken	1	6	61	244	40	32	3	2524	179
FSIS12036743	USA:Pennsylvania	2020	turkey	1	6	61	244	40	32	3	2524	179
FSIS12107699	USA:Arkansas	2021	chicken	1	6	61	244	40	32	3	2524	179
FSIS12214444	USA:Iowa	2022	cattle	1	6	61	176	40	32	3	4380	179
FSIS12320378	USA:Iowa	2023	chicken	1	6	61	176	40	32	3	4380	179
FSIS32206488	USA:Arkansas	2022	chicken	1	6	61	176	40	32	3	4380	179
FSIS32206495	USA:Arkansas	2022	chicken	1	6	61	176	40	32	3	4380	179

[a]: HHS_region 1 = (Connecticut, Maine, Massachusetts, New Hampshire, Rhode Island, Vermont)
HHS_region 2 = (New Jersey, New York, Puerto Rico, U.S. Virgin Islands)
HHS_region 3 = (Delaware, Maryland, Pennsylvania, Virginia, West Virginia, Washington D.C.)
HHS_region 4 = (Alabama, Florida, Georgia, Kentucky, Mississippi, North Carolina, South Carolina, Tennessee)
HHS_region 5 = (Illinois, Indiana, Michigan, Minnesota, Ohio, Wisconsin)
HHS_region 8 = (Colorado, Montana, North Dakota, South Dakota, Utah, Wyoming)

[b]: NA, not assigned

Shaded columns indicate shared alleles with strains SKBC9 and SKBC25.
