# Supplementary material for: American black bear (Ursus americanus) as a potential host for Campylobacter jejuni
Source: PLoS One. 2025 Sep 9;20(9):e0331559. doi: 10.1371/journal.pone.0331559 (PMC12419602; doi:10.1371/journal.pone.0331559)
Supplement: S3 Table — (PDF) [file pone.0331559.s003.pdf]

Supplementary Table 3: Strains in PubMLST with CC ST-682

Strain	Location	year	source	*aspA*	*glnA*	*gltA*	*glyA*	*pgm*	*tkt*	*uncA*	ST
78965	UK	1994	sand (bathing beach)	39	5	9	4	8	46	21	175
78970	UK	1994	sand (bathing beach)	37	2	9	2	8	46	23	176
87035	UK	1994	sand (bathing beach)	26	2	9	51	8	46	5	208
78966	UK	1994	sand (bathing beach)	2	2	9	51	8	46	5	215
WB CL95004 c16.7.02	UK	2002	starling	26	2	9	51	8	46	21	682
WB CL95011 c16.7.02	UK	2002	starling	17	2	9	51	121	46	21	686
WB CL95012 c16.7.02	UK	2002	starling	26	43	9	101	121	46	21	687
starling 4	UK: Oxfordshire	2002	starling feces	26	2	9	51	8	46	21	682
starling 11	UK: Oxfordshire	2002	starling feces	17	2	9	51	121	46	21	686
starling 12	UK: Oxfordshire	2002	starling feces	26	43	9	101	121	46	21	687
WB CL95003 c16.7.02	UK: Oxfordshire	2002	starling	35	43	9	5	8	46	21	681
WB CL95037e c6.2.03	UK: Wytham	2003	starling	35	2	8	51	121	46	21	818
EX114	UK	1997	broiler environment	4	2	9	5	8	46	21	914
CW04619 c15.6.04	UK: Oxfordshire	2004	starling	26	2	8	51	8	46	1	1019
CW04620 c15.6.04	UK: Oxfordshire	2004	starling	26	43	9	101	8	46	21	1020
WB CW04656 c15.6.04	UK	2004	starling	17	2	9	5	8	46	21	1021
WB CW04658 c16.6.04	UK: Oxfordshire	2004	starling	26	43	9	51	121	46	21	1022
ct86869 c4.6.04	UK: Wytham	2004	starling	26	2	9	51	8	2	21	1385
ct86858 c4.6.04	UK: Wytham	2004	starling	26	2	9	101	8	46	21	1386
ct86880 c8.6.04	UK: Wytham	2004	starling	35	43	9	51	8	2	21	1387
cw04658 c27.6.04	UK: Wytham	2004	starling	26	43	9	51	8	46	21	1390
ct43856 c26.1.04	UK: Wytham	2004	starling	26	43	9	2	8	46	21	1391
cw04605 c25.6.04	UK: Wytham	2004	starling	26	2	9	5	8	46	21	1392
cw04692 c22.6.04	UK: Wytham	2004	starling	26	2	9	51	121	46	21	1542
ct86898	UK	2004	starling	26	43	9	5	121	46	21	1503
CW04629	UK	2004	starling	17	2	8	51	121	46	21	1505
CW04707	UK	2004	starling	17	5	9	101	8	46	21	1507
cw04717	UK	2004	starling	26	2	9	51	121	46	21	1542
7423	UK: Grampian	2006	wild bird	35	2	9	51	8	2	21	1027
WB CT43886 c26.1.4	UK	2004	starling	26	43	9	2	8	46	21	1391
cl95027c c2.6.2003	UK	2003	starling	26	2	9	51	8	46	21	682
WB CT43816 r11.12.03	UK	2003	starling	35	2	9	51	8	2	21	1027
WB CL95027p c6.2.03	UK	2003	starling	26	2	9	51	8	46	21	682
WB CT86845 c25.5.04	UK	2004	starling	26	43	9	101	8	46	21	1020

WB CT86846 c25.5.04	UK	2004	starling	26	43	9	101	8	46	21	1020
WB CT86848 c25.5.04	UK	2004	starling	26	2	9	51	8	46	21	682
WB CT86849 c25.5.04	UK	2004	starling	26	2	9	51	8	46	21	682
WB CT86851 c2.6.04	UK	2004	starling	26	43	9	101	8	46	21	1020
WB CT86854 c2.6.04	UK	2004	starling	26	43	9	101	8	46	21	1020
WB CL95083 c2.6.04	UK	2004	starling	26	43	9	101	8	46	21	1020
WB CT86870 c4.6.04	UK	2004	starling	26	2	9	51	8	46	21	682
WB CT86874 c4.6.04	UK	2004	starling	26	43	9	101	8	46	21	1020
WB CL95044 c4.6.04	UK	2004	starling	26	43	9	101	8	46	21	1020
WB CL95059 c4.6.04	UK	2004	starling	26	43	9	101	8	46	21	1020
WB CL95083 c4.6.04	UK	2004	starling	26	43	9	101	8	46	21	1020
WB CT86791 c4.6.04	UK	2004	starling	26	43	9	101	8	46	21	1020
WB CT86854 c4.6.04	UK	2004	starling	26	43	9	101	8	46	21	1020
WB CT86858 c4.6.04	UK	2004	starling	26	2	9	101	8	46	21	1386
WB CT86878 c8.6.04	UK	2004	starling	35	2	9	51	8	2	21	1027
WB CT86879 c8.6.04	UK	2004	starling	35	2	9	51	8	2	21	1027
WB CT86882 c8.6.04	UK	2004	starling	26	43	9	101	8	46	21	1020
WB CT86883 c8.6.04	UK	2004	starling	26	43	9	101	8	46	21	1020
WB CT86888 c10.6.04	UK	2004	starling	26	43	9	101	8	46	21	1020
WB CW04604 c11.6.04	UK	2004	starling	26	43	9	101	8	46	21	1020
WB CW04607 c11.6.04	UK	2004	starling	26	43	9	101	8	46	21	1020
WB CW04608 c11.6.04	UK	2004	starling	26	43	9	101	8	46	21	1020
WB CW04610 c11.6.04	UK	2004	starling	26	43	9	101	8	46	21	1020
WB CW04612 c11.6.04	UK	2004	starling	26	43	9	101	8	46	21	1020
WB CW04613 c11.6.04	UK	2004	starling	26	43	9	101	8	46	21	1020
WB CW04614 c11.6.04	UK	2004	starling	35	2	8	51	121	46	21	818
WB CT86761 c11.6.04	UK	2004	starling	26	43	9	101	8	46	21	1020
WB CT86879 c11.6.04	UK	2004	starling	35	2	8	51	121	46	21	818
WB CT86880 c11.6.04	UK	2004	starling	35	2	9	51	8	2	21	1027
WB GR/P c15.6.04	UK	2004	starling	35	2	8	51	121	46	21	818
WB CW04616 c15.6.04	UK	2004	starling	35	2	8	51	121	46	21	818
WB CW04626 c15.6.04	UK	2004	starling	26	43	9	101	8	46	21	1020
WB CW04628 c15.6.04	UK	2004	starling	26	43	9	101	8	46	21	1020
WB CW04630 c15.6.04	UK	2004	starling	26	43	9	101	8	46	21	1020
WB CW04636 c15.6.04	UK	2004	starling	17	2	9	51	121	46	21	686
WB CW04638 c15.6.04	UK	2004	starling	26	43	9	101	8	46	21	1020
WB CW04639 c15.6.04	UK	2004	starling	35	2	8	51	121	46	21	818
WB CW04644 c15.6.04	UK	2004	starling	26	43	9	101	8	46	21	1020

WB CW04646 c15.6.04	UK	2004	starling	17	2	9	51	121	46	21	686
WB CW04648 c15.6.04	UK	2004	starling	26	43	9	101	8	46	21	1020
WB CW04651 c15.6.04	UK	2004	starling	17	2	9	51	121	46	21	686
WB CW04652 c15.6.04	UK	2004	starling	35	2	8	51	121	46	21	818
WB CW04653 c15.6.04	UK	2004	starling	17	2	9	51	121	46	21	686
WB CT43821 c15.6.04	UK	2004	starling	17	2	9	51	121	46	21	686
WB CT86804 c15.6.04	UK	2004	starling	26	43	9	101	8	46	21	1020
WB CT86858 c15.6.04	UK	2004	starling	26	43	9	101	8	46	21	1020
WB CW04659 c16.6.04	UK	2004	starling	26	43	9	101	8	46	21	1020
WB CW04662 c16.6.04	UK	2004	starling	26	43	9	101	8	46	21	1020
WB CW04663 c16.6.04	UK	2004	starling	26	43	9	5	121	46	21	1503
WB CW04666 c16.6.04	UK	2004	starling	26	43	9	101	8	46	21	1020
WB CT86780 c16.6.04	UK	2004	starling	26	43	9	101	8	46	21	1020
WB CT86819 c16.6.04	UK	2004	starling	17	2	9	51	121	46	21	686
WB CT86835 c16.6.04	UK	2004	starling	26	43	9	101	8	46	21	1020
WB CT86852 c16.6.04	UK	2004	starling	26	43	9	101	8	46	21	1020
WB CW04676 c18.6.04	UK	2004	starling	26	43	9	101	8	46	21	1020
WB CW04678 c18.6.04	UK	2004	starling	35	2	8	51	121	46	21	818
WB CW04679 c18.6.04	UK	2004	starling	26	43	9	101	8	46	21	1020
WB CW04681 c18.6.04	UK	2004	starling	17	2	9	51	121	46	21	686
WB CW04682 c18.6.04	UK	2004	starling	17	2	9	5	8	46	21	1021
WB CW04683 c18.6.04	UK	2004	starling	17	2	9	51	121	46	21	686
WB CW04684 c18.6.04	UK	2004	starling	35	2	9	51	8	2	21	1027
WB CW04686 c18.6.04	UK	2004	starling	17	2	9	51	121	46	21	686
WB CT86780 c18.6.04	UK	2004	starling	26	43	9	101	8	46	21	1020
WB CT86851 c18.6.04	UK	2004	starling	17	2	9	5	8	46	21	1021
WB CT86880 c18.6.04	UK	2004	starling	26	43	9	101	8	46	21	1020
WB CW04602 c18.6.04	UK	2004	starling	26	43	9	101	8	46	21	1020
WB CW04656 c18.6.04	UK	2004	starling	26	43	9	101	8	46	21	1020
WB CW04688 c22.6.04	UK	2004	starling	35	2	9	51	8	2	21	1027
WB CW04689 c22.6.04	UK	2004	starling	26	43	9	101	8	46	21	1020
WB CW04692 c22.6.04	UK	2004	starling	26	2	9	51	121	46	21	1542
WB CW04693 c22.6.04	UK	2004	starling	35	2	9	51	8	2	21	1027
WB CW04696 c22.6.04	UK	2004	starling	26	43	9	101	8	46	21	1020
WB CW04697 c22.6.04	UK	2004	starling	35	2	8	51	121	46	21	818
WB CW04698 c22.6.04	UK	2004	starling	26	43	9	101	8	46	21	1020
WB CW04701 c22.6.04	UK	2004	starling	26	43	9	101	8	46	21	1020
WB CW04702 c22.6.04	UK	2004	starling	26	43	9	101	8	46	21	1020

WB CW04706 c22.6.04	UK	2004	starling	26	43	9	101	8	46	21	1020
CW04772 c24.11.04	UK	2004	starling	26	43	9	51	121	46	21	1022
CW04774 c24.11.04	UK	2004	starling	26	43	9	51	121	46	21	1022
CW04776 c24.11.04	UK	2004	starling	26	43	9	51	121	46	21	1022
CW04780 c24.11.04	UK	2004	starling	26	43	9	51	121	46	21	1022
CW04785 c26.11.04	UK	2004	starling	26	43	9	51	121	46	21	1022
CW04789 c26.11.04	UK	2004	starling	26	43	9	51	121	46	21	1022
CW04791 c30.11.04	UK	2004	starling	26	43	9	51	121	46	21	1022
CW10311 c2.12.04	UK	2004	starling	35	2	9	51	8	2	21	1027
CW04772 c26.11.04	UK	2004	starling	26	43	9	51	121	46	21	1022
CW04779 c26.11.04	UK	2004	starling	26	43	9	51	121	46	21	1022
CL95078 c30.11.04	UK	2004	starling	26	43	9	51	121	46	21	1022
CL95078 c1.12.04	UK	2004	starling	26	43	9	51	121	46	21	1022
CW04790 c9.12.04	UK	2004	starling	26	43	9	51	121	46	21	1022
CW04605 c11.6.04	UK	2004	starling	26	43	9	101	8	46	21	1020
CW04609 c11.6.04	UK	2004	starling	26	2	9	51	8	46	21	682
WB CW04683 c22.6.04	UK	2004	starling	35	2	9	51	8	2	21	1027
WB CW04710 c24.6.04	UK	2004	starling	17	2	9	51	121	46	21	686
WB CW04712 c24.6.04	UK	2004	starling	26	43	9	101	8	46	21	1020
WB CW04714 c24.6.04	UK	2004	starling	26	43	9	101	8	46	21	1020
WB CW04716 c24.6.04	UK	2004	starling	26	43	9	101	8	46	21	1020
WB CW04718 c24.6.04	UK	2004	starling	35	2	8	51	121	46	21	818
WB CW04721 c24.6.04	UK	2004	starling	26	43	9	101	8	46	21	1020
WB CW04722 c24.6.04	UK	2004	starling	17	2	9	5	8	46	21	1021
WB CW04723 c24.6.04	UK	2004	starling	26	43	9	101	8	46	21	1020
WB CW04728 c24.6.04	UK	2004	starling	17	2	9	51	121	46	21	686
WB CW04696 c24.6.04	UK	2004	starling	26	43	9	101	8	46	21	1020
WB CW04731 c25.6.04	UK	2004	starling	35	2	8	51	121	46	21	818
WB CW04735 c25.6.04	UK	2004	starling	26	43	9	101	8	46	21	1020
WB CW04736 c25.6.04	UK	2004	starling	26	43	9	101	8	46	21	1020
WB CT86776 c25.6.04	UK	2004	starling	17	2	9	51	121	46	21	686
WB CT86897 c25.6.04	UK	2004	starling	17	2	9	51	121	46	21	686
WB CW04605 c25.6.04	UK	2004	starling	26	2	9	5	8	46	21	1392
WB CW04609 c25.6.04	UK	2004	starling	17	2	9	51	121	46	21	686
WB CW04658 c25.6.04	UK	2004	starling	35	2	9	51	8	2	21	1027
WB CW04712 c25.6.04	UK	2004	starling	26	43	9	101	8	46	21	1020
WB CW04713 c25.6.04	UK	2004	starling	35	2	9	51	8	2	21	1027
WB CW04714 c25.6.04	UK	2004	starling	26	43	9	101	8	46	21	1020

WB CW04704 c29.6.04	UK	2004	starling	26	43	9	101	8	46	21	1020
WB CW04714 c29.6.04	UK	2004	starling	35	2	9	51	8	2	21	1027
WB CW04683 c30.6.04	UK	2004	starling	35	2	9	51	8	2	21	1027
007A-0838	Canada: Quebec	2006	environmental water	26	2	9	51	8	46	1	4203
PIK3	Finland	2005	Unk.	17	2	8	51	8	46	21	4573
VDL7103	USA: Iowa	2008	sheep	35	2	9	432	8	46	21	5128
MIDO 818 (5)	USA: Georgia	2005	environmental water	35	2	9	51	8	2	21	1027
NORO 516 (12)	USA: Georgia	2005	environmental water	26	2	9	51	8	46	21	682
MIDO 301 (13)	USA: Georgia	2005	environmental water	26	2	9	51	8	46	21	682
MIDO 812 (78)	USA: Georgia	2005	environmental water	35	2	9	51	8	2	21	1027
CSSS_66821	UK: Nottinghamshire	2002	human: unspecified	26	2	9	51	8	46	21	682
007A-0377	Canada: Quebec	2005	environmental water	35	2	9	51	8	2	21	1027
007A-0385	Canada: Quebec	2005	environmental water	26	2	9	51	8	46	21	682
007A-0391	Canada: Quebec	2005	environmental water	26	2	9	51	8	46	21	682
007A-0792	Canada: Quebec	2006	environmental water	26	2	9	51	8	46	21	682
007A-0828	Canada: Quebec	2006	environmental water	26	2	9	51	8	46	21	682
06_BB_25	Australia	2006	blackbird	35	2	9	51	8	46	21	3068
jonas_star_3	Sweden	2000	starling	26	2	9	51	8	46	21	682
jonas_star_7	Sweden	2000	starling	26	2	9	51	8	46	21	682
jonas_star_5	Sweden	2000	starling	26	2	9	51	8	46	21	682
jonas_star_1	Sweden	2000	starling	26	2	9	51	8	46	21	682
2011D-8859	USA	Unk.	Unk.	35	2	9	534	8	46	21	6677
J-Br-706	USA: Ohio	2010	starling	26	43	9	101	8	46	21	1020
E-Br-2	USA: Ohio	2010	starling	26	43	9	101	8	46	21	1020
I-Br-15	USA: Ohio	2010	starling	26	43	9	101	8	46	21	1020
I-Br-14	USA: Ohio	2010	starling	26	43	9	101	8	46	21	1020
H-Br-7	USA: Ohio	2010	starling	17	2	9	5	8	46	21	1021
LB_BS3.1_21C2	Thailand: Bangkok	2012	broiler environment	35	2	9	5	8	553	21	6995
E120455	Luxembourg	2012	environmental water	17	2	9	5	8	46	21	1021
W0004	UK: Scotland	2015	wild bird	26	2	9	51	8	46	21	682
BS3.121C2	Thailand	2012	broiler environment	35	2	9	5	8	553	21	6995
B1432b	New Zealand: Manawatu	2009	wild bird	35	43	9	5	8	46	21	681
B1624b	New Zealand: Manawatu	2009	wild bird	26	2	9	51	8	46	5	208
W860b	New Zealand: Waikato	2013	environmental water	26	2	9	51	8	46	21	682
CL95044_4.6.4	UK	Unk.	starling	26	43	9	101	8	46	21	1020
starling1020	UK	Unk.	starling	26	43	9	101	8	46	21	1020
SGEHI2013-C591-1	Singapore	2013	wild bird	35	43	8	51	8	46	21	9603
PNUSAC005563	USA	Unk.	Unk.	26	2	9	51	8	46	21	682

PNUSAC005518	USA	Unk.	Unk.	26	2	9	51	8	46	21	682
FSIS11920083	USA: California	2019	chicken offal or meat	35	2	9	432	8	46	21	5128
FSIS1607521	USA: California	2016	cattle	35	2	8	51	121	46	21	818
FSIS11813822	USA: Minnesota	2018	cattle	26	2	9	51	8	46	21	682
**SKBC94**	**USA: North Carolina**	**2016**	**black bear**	26	2	9	51	8	46	21	682
125	UK	Unk.	starling	26	43	9	101	8	46	21	1020
10352	USA	Unk.	starling	35	2	281	5	8	46	21	12448
CMB210274	New Zealand	2021	environmental water	237	2	9	5	8	222	21	11295
CMB210352	New Zealand	2021	environmental water	237	2	9	5	8	222	21	11295
B203-270820-01	Luxembourg	2020	wild bird	4	43	9	5	8	46	21	11385
FSIS12106873	USA	2021	calf	26	2	9	51	8	46	21	682
OXCBB-3596	UK	2004	chicken	26	43	9	51	121	46	21	1022
OXCBB-3684	UK	2004	chicken	26	43	9	51	121	46	21	1022
OXCBB-3577	UK	2004	chicken	26	43	9	51	121	46	21	1022
OXCBB-3478	UK	2004	chicken	26	43	9	51	121	46	21	1022
OXCBB-3510	UK	2004	chicken	26	43	9	51	121	46	21	1022
OXCstar152	UK	2004	chicken	26	43	9	51	121	46	21	1022
W69	Thailand	2018	environmental water	26	2	9	51	8	46	21	682
