# Supplementary material for: American black bear (Ursus americanus) as a potential host for Campylobacter jejuni
Source: PLoS One. 2025 Sep 9;20(9):e0331559. doi: 10.1371/journal.pone.0331559 (PMC12419602; doi:10.1371/journal.pone.0331559)
Supplement: S4 Table — (PDF) [file pone.0331559.s004.pdf]

Supplementary Table 4: *C. jejuni* strains in PubMLST that share alleles with either SKBC3 or SKBC5

Strain	Prov/State/Region	Year	source	aspA	glnA	gltA	glyA	pgm	tkt	uncA	ST	clonal complex
007A-0615	Quebec Canada	2006	environmental waters	255	339	279	383	479	391	276	4361	
VDL26018	Iowa, USA	2011	dog	305	6	61	176	40	180	3	6971	
SFBRC-47	Georgia, USA	2013	environmental waters	305	409	339	664	578	391	344	7945	
SFBRC-61	Georgia, USA	2013	environmental waters	305	6	61	176	40	180	224	7943	
PNUSAC007623	NA	NA	NA	305	6	137	176	40	32	3	10539	ST-179 complex
PNUSAC006289	HHS Region 5, USA	2018	human	305	6	61	176	40	180	3	6971	
PNUSAC006563	HHS Region 8, USA	2018	human	305	6	61	176	40	180	3	6971	
SKBC3	North Carolina, USA	2014	black bear	255	530	279	607	740	585	276	7630	
SKBC5	North Carolina, USA	2014	black bear	305	756	279	607	479	585	276	10620	
SFBRC-36	Georgia, USA	2013	environmental waters	305	6	61	176	40	180	224	7943	
SFBRC-59	Georgia, USA	2013	environmental waters	305	6	61	176	40	180	224	7943	
SFBRC-60	Georgia, USA	2013	environmental waters	305	6	61	176	40	180	224	7943	
SFBRC-66	Georgia, USA	2013	environmental waters	426	530	339	607	779	391	344	7949	

Alleles from SKBC3 or SKBC5 highlighted in orange are present in more than one strain, while those highlighte in yellow were present in only the particular isolate.
