# Supplementary material for: American black bear (Ursus americanus) as a potential host for Campylobacter jejuni
Source: PLoS One. 2025 Sep 9;20(9):e0331559. doi: 10.1371/journal.pone.0331559 (PMC12419602; doi:10.1371/journal.pone.0331559)
Supplement: S5 Table — (PDF) [file pone.0331559.s005.pdf]

Supplementary Table 5: Nucleotide identity of CJIE1 (Mu-like) genes from *C. jejuni* isolated from black bears to a subset of RM1221 CJIE1 genes.

		**Strain** (Location)					
**RM1221 locus tags**	**Description**	**SKBC1** (near *npdA*)	**SKBC3-1** (near *rarA*)	**SKBC3-2** (near *ctsT*)	**SKBC5-1** (in CJIE2-like)	**SKBC5-2** (in CJIE3-like)	**SKBC25** (near *selU*)
CJE0270	DNA transposition protein A	**44.0%**	**90.9%**	**44.7%**	**90.1%**	**43.7%**	**86.2%**
CJE0269	DNA transposition protein B	**46.9%**	**95.7%**	**46.4%**	**95.7%**	**46.4%**	**95.8%**
CJE0265	host-nuclease inhibitor protein Gam	**92.4%**	**89.6%**	**94.0%**	**89.6%**	**94.0%**	**90.9%**
CJE0256	*dns* (extracellular DNase)	**-**[A]	**92.9%**	**-**	**92.9%**	**-**	**99.0%**
CJE0254	tail D protein	**57.9%**	**96.6%**	**58.1%**	**96.6%**	**58.1%**	**97.6%**
CJE0252	tail protein	**70.0%**	**96.8%**	**69.9%**	**96.8%**	**69.9%**	**99.7%**
CJE0251	Mu-like prohage F protein	**-**	**95.7%**	**-**	**95.7%**	**-**	**99.6%**
CJE0244	Mu-like prohage I protein	**-**	**95.7%**	**-**	**95.7%**	**-**	**99.6%**
CJE0236	baseplate assembly protein V	**97.4%**	**97.4%**	**97.4%**	**97.2%**	**97.4%**	**97.9%**
CJE0235	baseplate assembly protein W	**98.6%**	**92.1%**	**92.1%**	**92.1%**	**90.7%**	**97.9%**
CJE0233	baseplate assembly protein J	**97.1%**	**94.7%**	**94.7%**	**94.7%**	**94.9%**	**96.1%**
CJE0232	tail protein	**96.9%**	**96.8%**	**94.2%**	**96.8%**	**95.8%**	**96.8%**
CJE0231	tail fiber H protein	**94.2%**	**96.8%**	**96.8%**	**96.8%**	**96.9%**	**95.8%**
CJE0227	major tail sheath protein	**97.8%**	**95.4%**	**95.5%**	**95.7%**	**95.8%**	**97.9%**
CJE0226	major tail tube protein	**71.5%**	**96.5%**	**71.5%**	**96.7%**	**71.5%**	**96.5%**
CJE0222	tail tape measure protein	**42.40%**	**77.4%**	**42.60%**	**77.4%**	**42.60%**	**77.6%**

The nucleotide identity of CJIE1 genes from *C. jejuni* strain RM1221 to CJIE1 from strain SKBC1, two different CJIE1 loci in strainSKBC3, two different CJEI1 loci in strain SKBC5, and CJIE1 in strain SKBC25. Identities above 80% are shaded blue. Identities below 80% are shaded red.

[A]: - denotes the absence of the gene.
