# Supplementary material for: American black bear (Ursus americanus) as a potential host for Campylobacter jejuni
Source: PLoS One. 2025 Sep 9;20(9):e0331559. doi: 10.1371/journal.pone.0331559 (PMC12419602; doi:10.1371/journal.pone.0331559)
Supplement: S1 Fig — (PDF) [file pone.0331559.s006.pdf]

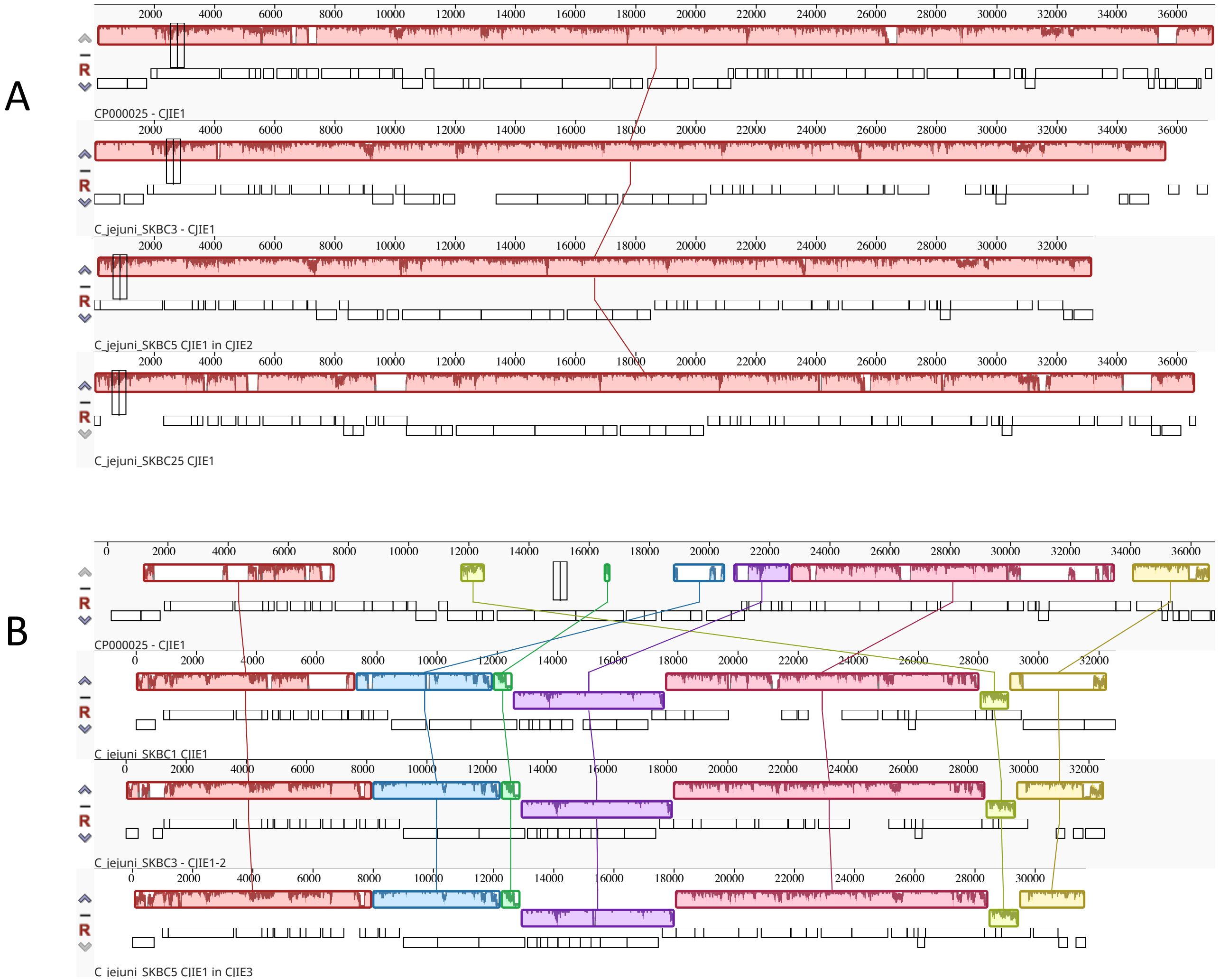

Supplementary Figure 1. Genome alignment of CJIE1 (Mu-like bacteriophages). Genome alignment of CJIE1-like genomes from *Campylobacter jejuni* strain RM1221 and the bear isolates with CJIE1-like bacteriophages with similar transposase-encoding genes (A) using Mauve revealed one collinear block conserved among bacteriophage genomes disrupted by insertions and deletions. Genome alignment of CJIE1-like genomes from *C. jejuni* strain RM1221 and the bear isolates with CJIE1-like bacteriophages with dissimilar transposase-encoding genes (B) revealed seven collinear blocks conserved among bacteriophage genomes disrupted by insertions and deletions. Each bacteriophage genome is arranged horizontally and homologous blocks in each genome are shown as identically colored regions linked across the Mu-like bacteriophages. Conserved blocks that were inverted compared to RM1221 in the figure are located beneath the CJIE1-like bacteriophage genome. The order of Mu-like bacteriophages with similar transposase-encoding genes to RM1221 (A) is: RM1221, SKBC3, SKBC5 and SKBC25. The order of Mu-like bacteriophages with dissimilar transposase-encoding genes to RM1221 (B) is: RM1221, SKBC1, SKBC3, and SKBC5.
