# Supplementary material for: American black bear (Ursus americanus) as a potential host for Campylobacter jejuni
Source: PLoS One. 2025 Sep 9;20(9):e0331559. doi: 10.1371/journal.pone.0331559 (PMC12419602; doi:10.1371/journal.pone.0331559)
Supplement: S2 Fig — (PDF) [file pone.0331559.s007.pdf]

A

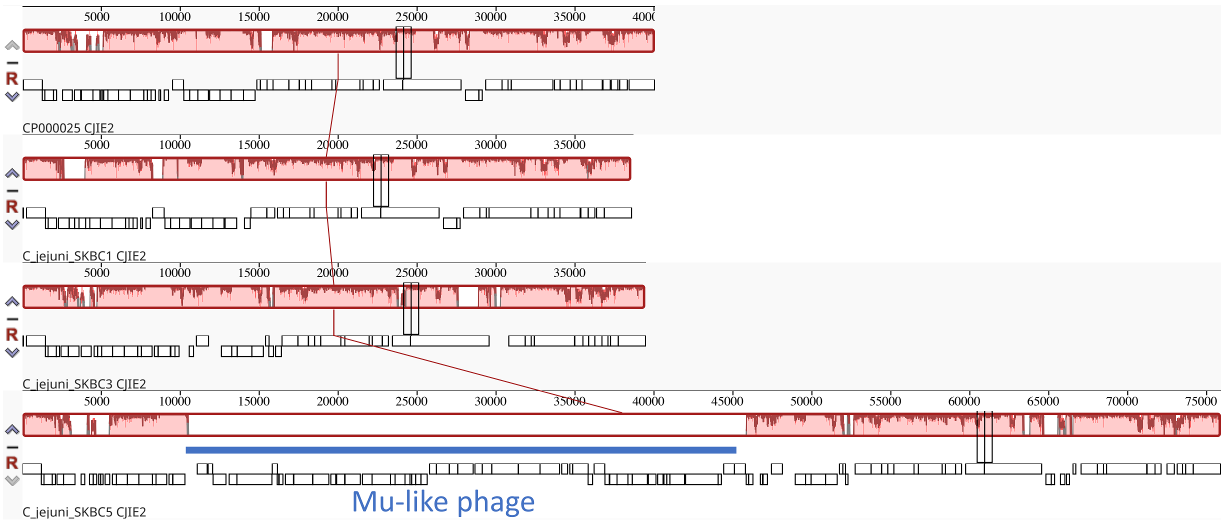

B

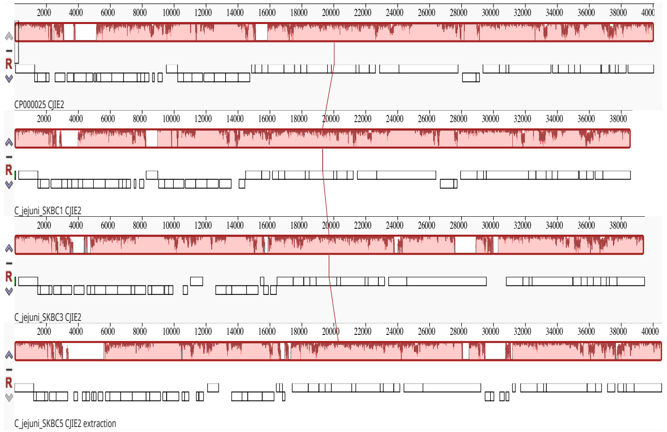

Supplementary Figure 2. Genome alignment of CJIE2. Genome alignment of CJIE2-like genomes from *C. jejuni* strain RM1221 and the *C. jejuni* isolates from bears using Mauve revealed one collinear block conserved among bacteriophage genomes disrupted by insertions and deletions (A). The order of CJIE2-like genomes is RM1221, SKBC1, SKBC3, and SKBC5. CJIE2 in SKBC5 possesses a Mu-like bacteriophage is underlined with a blue bar. This Mu-like bacteriophage element disrupts the collinearity of the CJIE2-like genomes. In (B), genome alignment of CJIE2-like genomes from *C. jejuni* strain RM1221 and the *C. jejuni* isolates from bears using Mauve, in which the Mu-like bacteriophage has been removed from the CJIE2-like bacteriophage of SKBC5.
