# Supplementary material for: American black bear (Ursus americanus) as a potential host for Campylobacter jejuni
Source: PLoS One. 2025 Sep 9;20(9):e0331559. doi: 10.1371/journal.pone.0331559 (PMC12419602; doi:10.1371/journal.pone.0331559)
Supplement: S3 Fig — (PDF) [file pone.0331559.s008.pdf]

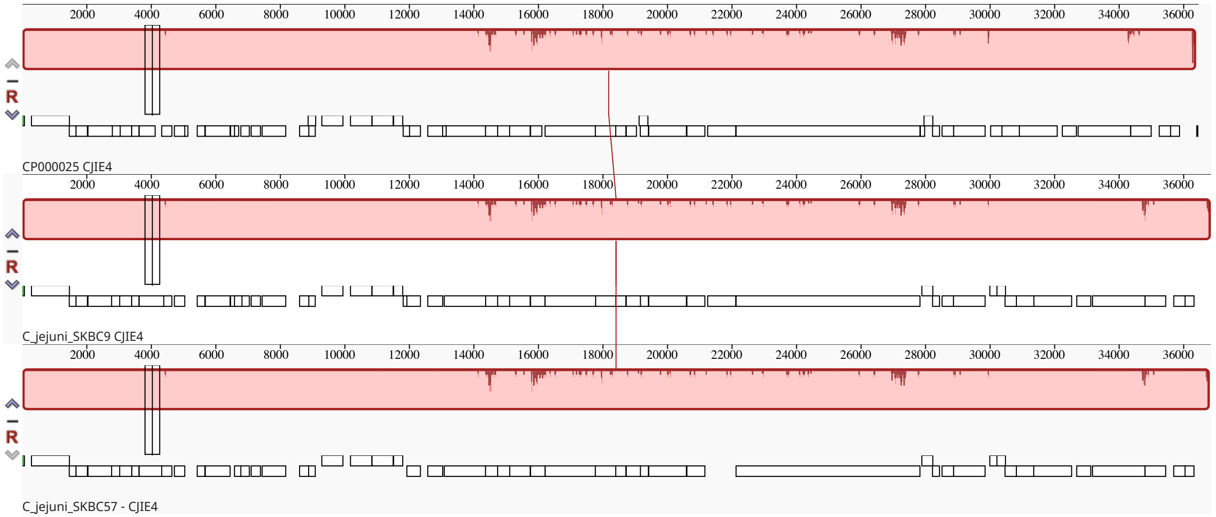

Supplementary Figure 3. Comparisons of CJIE4. Genome alignment of CJIE4 genomes from *C. jejuni* strain RM1221 and the *C. jejuni* isolates from bears using Mauve revealed one collinear block conserved among bacteriophage genomes with no large insertion or deletion disruptions. The order of aligned CJIE4 is: RM1221, SKBC9, and SKBC57.
