# Supplementary material for: American black bear (Ursus americanus) as a potential host for Campylobacter jejuni
Source: PLoS One. 2025 Sep 9;20(9):e0331559. doi: 10.1371/journal.pone.0331559 (PMC12419602; doi:10.1371/journal.pone.0331559)
Supplement: S4 Fig — (PDF) [file pone.0331559.s009.pdf]

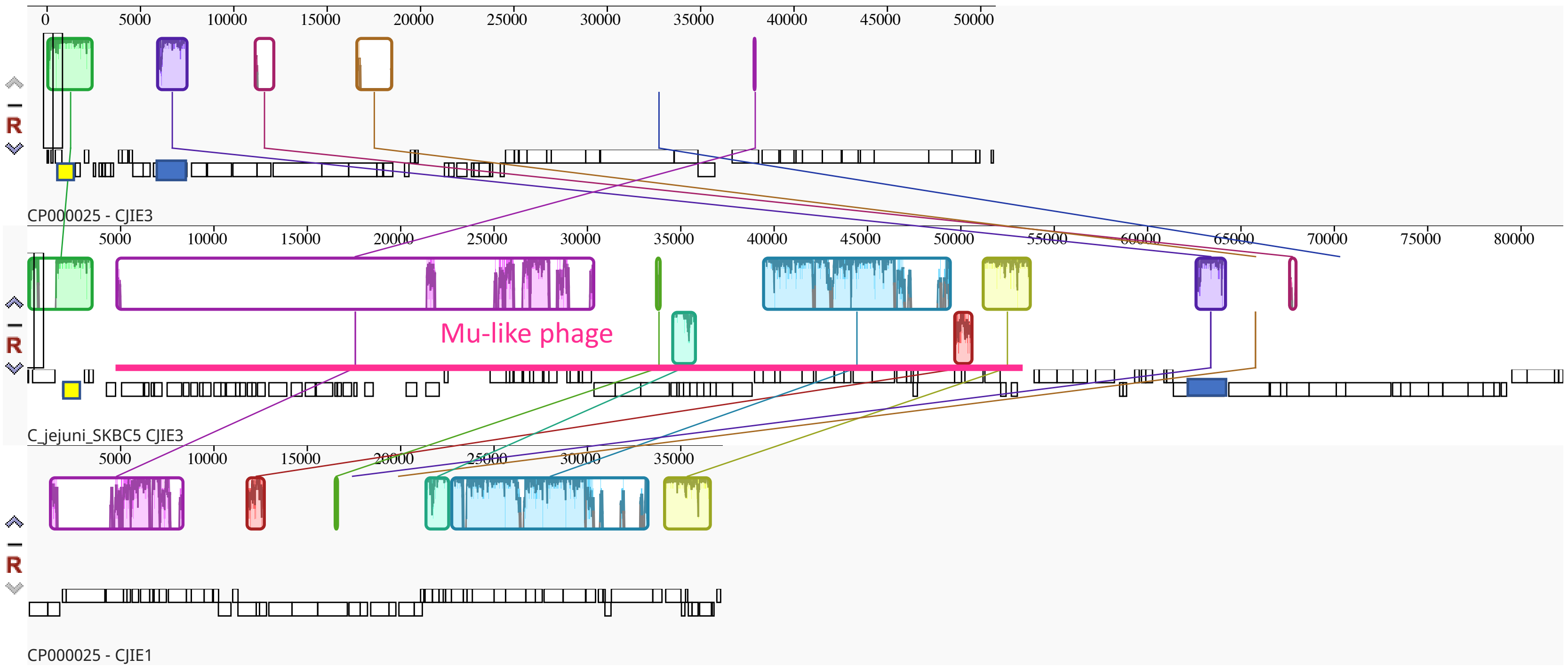

Supplementary Figure 4. Comparison of element at CJIE3 insertion site. Insertions and deletions were compared between CJIE3 in *C. jejuni* strain RM1221 and the element at the CJIE3 integration site at the tRNA-Arg gene near aroB and tgt in SKBC5. CJIE1 in *C. jejuni* strain RM1221 was included in the alignment with the order: RM1221 CJIE3, element in SKBC5 and RM1221 CJIE1. Alignment was created and visualized by Mauve software. Conserved blocks that were inverted compared to RM1221 in the figure are located beneath the SKBC5 integrated element. As mentioned, this element in SKBC5 possesses a Mu-like bacteriophage that is underlined with a pink bar. CJIE3 site specific-nuclease (integrase) and traG-like genes in both RM1221 and SKBC5 are colored yellow and blue, respectively.
