# Supplementary material for: American black bear (Ursus americanus) as a potential host for Campylobacter jejuni
Source: PLoS One. 2025 Sep 9;20(9):e0331559. doi: 10.1371/journal.pone.0331559 (PMC12419602; doi:10.1371/journal.pone.0331559)
Supplement: S5 Fig — (PDF) [file pone.0331559.s010.pdf]

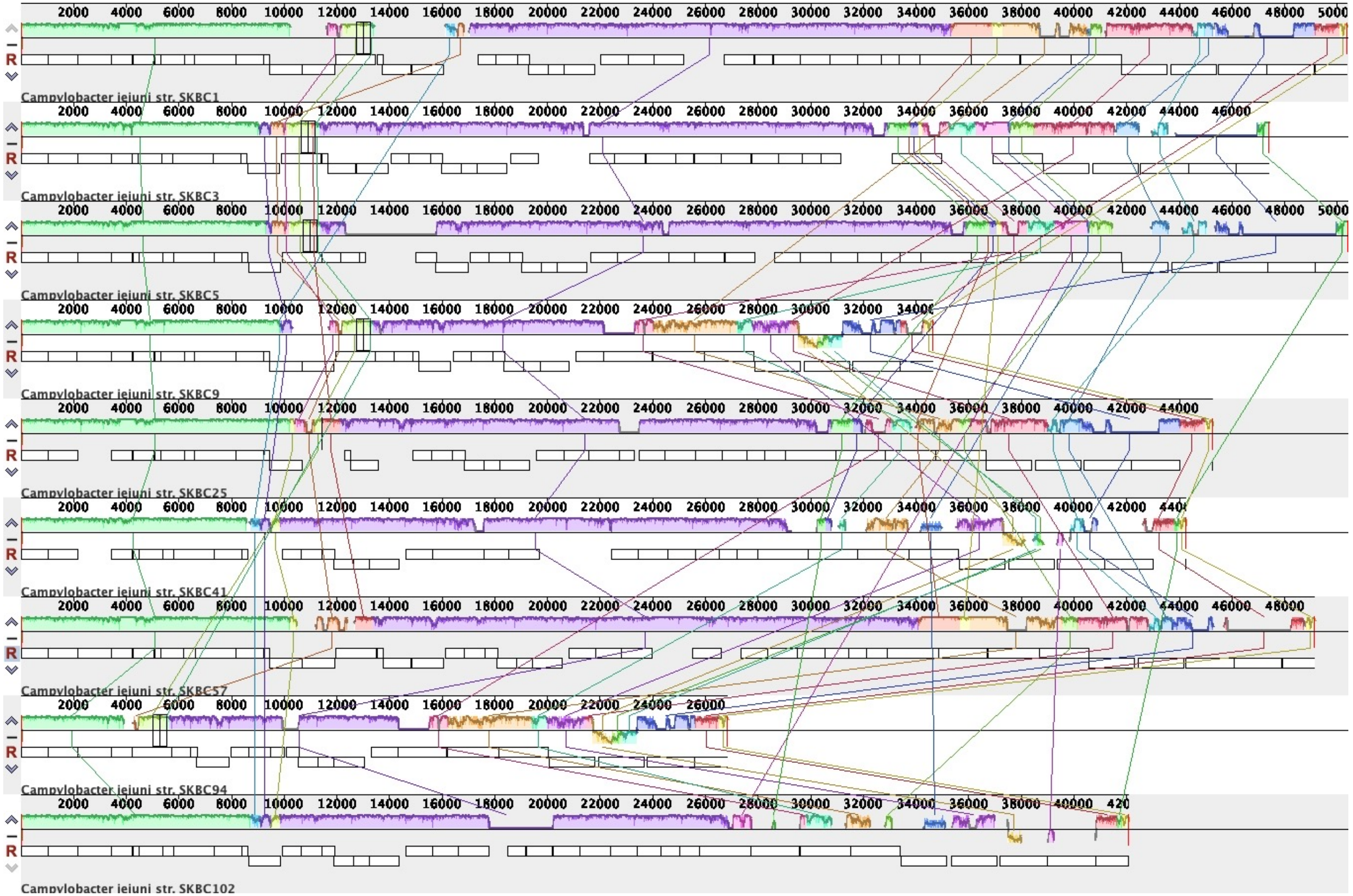

Supplementary Figure 5. Comparisons of flagellar modification loci. Flagellar modification (FM) biosynthesis loci alignment of the *C. jejuni* isolates from bears using Mauve revealed several local collinear blocks conserved among FM loci disrupted by insertions and deletions. The order of FM loci is SKBC1, SKBC3, SKBC5, SKBC9, SKBC25, SKBC41, SKBC57, SKBC94 and SKBC102. Conserved blocks that were inverted compared to SKBC1 or other previous strain in the figure are located beneath the FM loci.
