# Supplementary material for: American black bear (Ursus americanus) as a potential host for Campylobacter jejuni
Source: PLoS One. 2025 Sep 9;20(9):e0331559. doi: 10.1371/journal.pone.0331559 (PMC12419602; doi:10.1371/journal.pone.0331559)
Supplement: S6 Fig — (PDF) [file pone.0331559.s011.pdf]

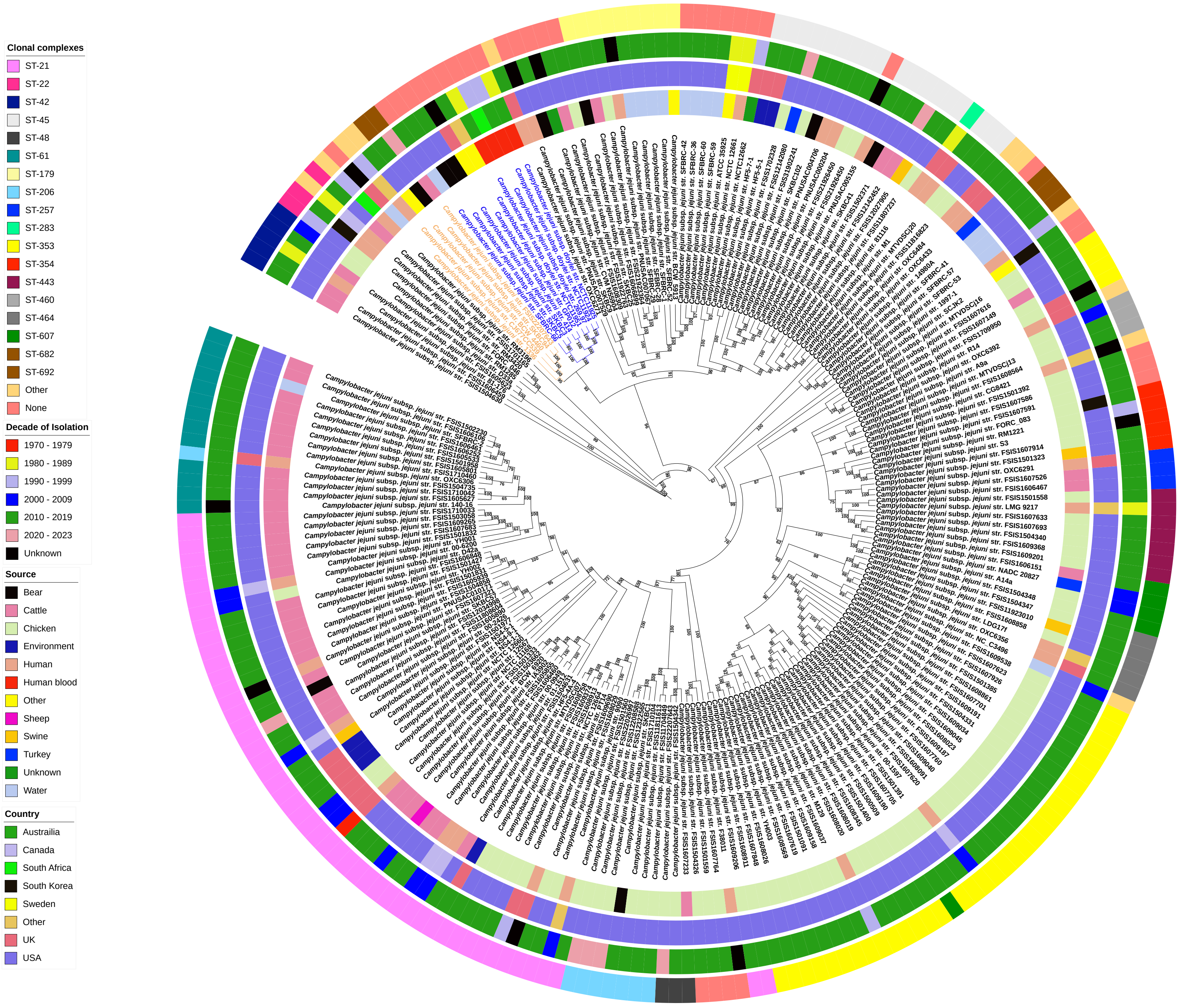

Supplemental Figure 6. *C. jejuni* isolates from bears (n=9) were compared with a global collection of publicly available genomes (n = 71) along with environmental and animal associated genomes isolated in the Southeastern U.S. (n=143). The 1,107 core genes from the genomes of all 223 genomic sequences were identified by Roary software and aligned using MAFFT. The dendrogram was constructed using RAxML with the GTRCAT model and 1,000 bootstraps. Clustered branches that are colored blue identify strains possessing deletions in both *cdt* and *mfr* loci, and those colored orange on adjacent branches possessing nonsense, point mutations in the *cdt* genes. Colored circles represent from inner to outer: (1) Source of isolation; (2) Country of isolation; (3) Decade of isolation; and (4) Clonal complex.
